# Stress Corrosion Cracking Susceptibility of 304L Substrate and 308L Weld Metal Exposed to a Salt Spray

**DOI:** 10.3390/ma10020187

**Published:** 2017-02-15

**Authors:** Chia-Hao Hsu, Tai-Cheng Chen, Rong-Tan Huang, Leu-Wen Tsay

**Affiliations:** 1 Institute of Materials Engineering, National Taiwan Ocean University, Keelung 20224, Taiwan; aa7788329@yahoo.com.tw (C.-H.H.); rthuang@mail.ntou.edu.tw (R.-T.H.); 2 Division of Nuclear Fuels and Materials, Institute of Nuclear Energy Research, Lungtan, Taoyuan 32546, Taiwan; tcchen@iner.gov.tw

**Keywords:** 308L deposit, 304L substrate, U-bend, weight loss, stress corrosion cracking

## Abstract

304 stainless steels (SS) were considered as the materials for a dry storage canister. In this study, ER (Electrode Rod) 308L was utilized as the filler metal for the groove and overlay welds of a 304L stainless steel substrate, which was prepared via a gas tungsten arc-welding process in multiple passes. The electron backscatter diffraction (EBSD) map was used to identify the inherent microstructures in distinct specimens. U-bend and weight-loss tests were conducted by testing the 304L substrates and welds in a salt spray containing 5 wt % NaCl at 80 °C to evaluate their susceptibility to stress corrosion cracking (SCC). Generally, the weight loss of the ER 308L deposit was higher than that of the 304L substrate in a salt spray in the same sample-prepared condition. The dissolution of the skeletal structure in the fusion zone (FZ) was responsible for a greater weight loss of the 308L deposit, especially for the cold-rolled and sensitized specimen. Cold rolling was detrimental and sensitization after cold rolling was very harmful to the SCC resistance of the 304L substrate and 308L deposit. Overall, the SCC susceptibility of each specimen was correlated with its weight loss in each group.

## 1. Introduction

AISI 304 stainless steel (SS) is one of the candidate materials used for dry storage canisters of spent nuclear fuel as an interim storage measure before final disposal [1,2]. A great concern for the long-term integrity of canisters located near the coastline is chloride-induced stress corrosion cracking of austenitic stainless steels [3,4,5]. The decay heat of the spent nuclear fuel in dry storage, by design, will dissipate through the stainless steel canister via natural convection. Therefore, the degradation of the 304 SS canister, which is heated to a maximum temperature below 180 °C by estimation, can be complicated by the stress corrosion cracking (SCC) that occurs in a chloride-containing environment. Therefore, it is of technological interest to evaluate the SCC susceptibility of 304 SS with the salt deposit [1] and salt spray [2,6].

304 SS is known to be prone to SCC in the presence of chloride [7,8,9,10,11,12,13]. Corrosion pits act as stress concentrators to initiate SCC in 304, 304L, and 316L SSs [4,14]. For deformed 304L SS, pitting is more likely to occur in the regions of high equivalent plastic strain [15]. The pitting susceptibility of 304L SS in 0.1 N NaCl solutions varies with degree of cold rolling. The pitting potential decreases with the cold reduction up to 50%, while it increases beyond 50% [16]. It has been reported that microvoids are more likely to be formed at the slip bands [17] and preferential dissolution of the slip bands increases the SCC susceptibility of cold-worked 304L SS at room temperature in 1 M HCl solution [12]. It is well known that metastable austenitic SSs may undergo austenite to martensite transformations under straining [18,19]. The induced martensite is beneficial in improving tensile properties [19], but harmful to SCC resistance [13,20]. Surface finishing operations, like machining and grinding, have led to a drastic decrease in the SCC resistance of 304L SS in chloride environments [13,20]. The cumulative effect of extensive grain fragmentation, martensitic transformation, increased surface roughness, and localized stresses accumulated at the surface asperities make the surface electrochemically much more active compared to the solution annealed condition [20]. Moreover, the induced martensite in cold-worked 304L SS causes low-temperature sensitization at 500 °C [21,22,23] and increased susceptibility to SCC in a boiling water reactor (BWR)-simulated environment [21].

Hot cracking of austenitic SS welds can be avoided by the formation of a limited amount of δ ferrite in the fusion zone (FZ). However, the pitting corrosion resistance of austenitic SS welds will be affected by the δ-ferrite content in the FZ [24,25]. It has been established that the ferrite and ferrite-austenite interface in the FZ of a 304 weld are attacked preferentially in a 1 M NaCl + 0.5 M HCl solution [24]. The time required to break down the passive film of the 316 FZ decreases with increasing δ-ferrite content [25]. Furthermore, the time to fracture of 316L SS, determined by a slow strain-rate tensile test in a 1 N H_2_SO_4_ + 0.5 N NaCl solution, is reduced with increasing amounts of prior cold work if welding is applied [26]. The SCC resistance of the FZ for an autogenous 316 weld is inferior to its counterpart base metal in a 5 N H_2_SO_4_ + 0.5 N NaCl solution [27]. The higher ferrite contents of the FZ of 309, 309Mo, and 309Cb SSs, relative to that of 316 SS, considerably reduce the SCC resistance in a 5 N H_2_SO_4_ + 0.5 N NaCl solution [28]. With nearly the same δ-ferrite content, the SCC resistance of a 309LMo deposit is obviously higher than that of a 308L deposit in a salt spray [29].

Mechanically and/or thermally induced stresses are unavoidably introduced in the production of an SS canister. In this work, U-bend tests were conducted to evaluate the SCC susceptibility of 304L substrate and groove weld using ER (Electrode Rod) 308L as the filler metal in a salt spray. In addition, microstructural effects on the corrosion resistance of the 304L substrate and 308L deposit in the salt spray were determined based on the weight loss of the specimens. The selective dissolution of one of the duplex and/or multi-phases in the specimens may prompt crack initiation, thus activating SCC. Therefore, the SCC susceptibility of the specimens may be related to the results of weight loss tests.

## 2. Material and Experimental Procedures

### 2.1. Preparation of Samples

The dimensions of the test specimens, which were sectioned from groove and overlay welds using an electro-discharge wire cutter, have been presented in a previous work [29]. A 6-mm-thick AISI 304L steel plate grooved at an angle of 60° with a root opening of 2 mm was provided for the subsequent welding. A gas tungsten arc-welding process was employed to fill up the joint in three passes, using ER 308L as the filler metal. The chemical compositions (by weight percentage) of the 304L plate (base metal, BM) and the 308L filler metal were listed in Table 1. The groove weld in the as-welded condition was named the AW specimen. To investigate the effect of rolling on the microstructures and corrosion properties of the 304L substrate and groove weld, the samples were rolled to a final thickness of 4.8 mm (a 20% reduction in thickness) at room temperature; the corresponding specimens were referred to as the cold-rolled (CR) specimens. A sensitization treatment at 650 °C for 10 h was applied after cold rolling; these specimens were named the CRS specimens. In order to evaluate the corrosion resistance of the 308L deposit, the multi-pass weld overlays were applied on the 20-mm-thick 304L substrate using the gas tungsten arc-welding process. All weld-metal specimens were wire-cut from the overlay welds with two layers of deposits at a height of 4.5 mm. The corrosion resistance (weight loss) of the deposits in a salt spray was compared with that of the 304L substrate in the same sample-prepared conditions. The overlay weld used the same designation as the groove weld. The test matrix of the current study is shown in Table 2.

### 2.2. SCC and Weight-Loss Tests in a Salt Spray

A U-bend specimen with original dimensions of 120 (L) × 10 (W) × 2 mm (T) was tested in a salt spray consisting of 5 wt % NaCl at 80 °C. A 5% augmented strain could be applied to the U-bend specimen by imposing the specimen on a die block with a 20 mm radius. Before bending, the surfaces of the U-bend specimen were ground with 2000-grit abrasive paper. To facilitate the initiation and inspection of crack growth during the U-bend test, a hole with a diameter of 1.55 mm was drilled through the specimen using an electro-discharged machine. To measure the crack length, the U-bend specimens were periodically removed from the salt spray chamber and then inspected by using an SZ-STS stereo-microscope (Olympus, Tokyo, Japan) at 30× magnification. Before inspection, the surface of the covering on the U-bend specimen was removed with a soft brush and flashed by ethanol. The rust on the surface of the U-bend specimen was easily removed, revealing a shiny surface with hairline scratches. Only surface cracks of various specimens were measured and used to rank the specimens according to cracking susceptibility. The cracking susceptibility of the U-bend specimens was indexed by the maximum crack length (MCL, the longest crack length) and total crack length (TCL, summation of individual crack lengths). To confirm the overall tendency of cracking, the U-bend specimens after testing were sectioned into small samples and subjected to metallographic preparation for inspection of the crack features.

The welds for the weight-loss tests were sliced from the overlay welds parallel to the weld interface. To evaluate the corrosion resistance, the weight loss of all the weld-metal specimens, which had the dimensions of 10 (L) × 10 (W) × 3 mm (T), was assessed in the same salt spray as the U-bend specimens. After testing for 288 h, the weight loss divided by the exposed surface area of the specimen was recorded and compared with the 304L substrate. Both the U-bend and weight-loss test data shown in this work were the results of at least three samples.

### 2.3. Ferrite Determinations and Microstructural Observations

A Fischer FMP30 ferritescope (Helmut Fischer, Sindelfingen, Germany) was used to determine the ferrite contents or induced α′-martensite in the specimens [30]. The metallographic specimens were observed by BX51 optical microscope (OM, Olympus, Tokyo, Japan) and JSM-7100F field emission scanning electron microscope (SEM, JEOL, Tokyo, Japan). Microhardness of distinct specimens was obtained by MVK-G1500 Vickers hardness tester (Mitutoyo, Kawasaki, Japan) using a diamond pyramidal indenter with a 0.3 kgf load (HV0.3) and a 15 s dwell time. Moreover, the specimens were examined by a SEM equipped with NordlysMax^2^ electron backscatter diffraction (EBSD) detector (Oxford Instruments, Abingdon, UK) to identify the phase constitutions in the rolled steel plates and weld deposits. The fractographs of the U-bend specimens after salt spray tests were also examined by using a SEM. Furthermore, carbides that precipitated in the sensitized specimens were also characterized by a JEM-2000EX transmission electron microscope (TEM, JEOL, Tokyo, Japan) and X-Max^N^ energy dispersive spectroscopy (EDS) systems (Oxford Instruments, Abingdon, UK) attached to the SEM.

## 3. Results

### 3.1. Microstructures

**Figure 1:**
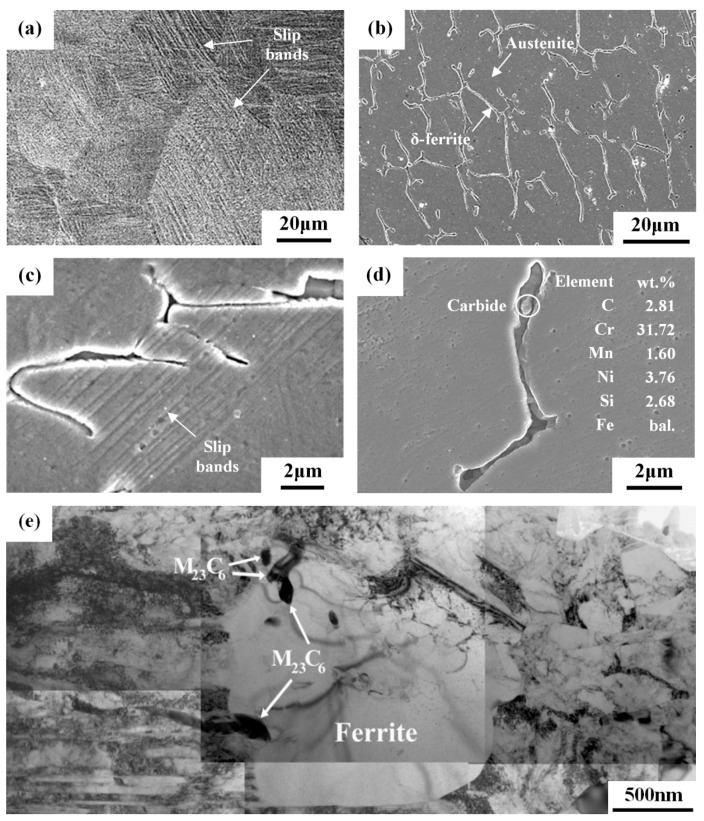
SEM micrographs of the (**a**) 304L-CR; (**b**) 308L-AW; (**c**) 308L-CR; (**d**) 308L-CRS specimens, and (**e**) TEM micrograph of 308L-CRS specimen.

Table 3 and Figure 1 show the hardness and microstructure of various specimens, respectively. The 304L base metal (304L-BM), which had a hardness of 165 HV0.3, revealed the microstructures of equiaxial grains with twins inside the austenite matrix. Rolling caused strain-hardening (340 HV0.3), which was accompanied by the introduction of slip bands and martensites in the specimen (Figure 1a). Sensitization treatment diminished the density of slip bands, hence, reducing the hardness to 278 HV0.3. The hardness of the as-welded FZ was similar to that of 304L-BM. In contrast, skeletal structures with vermicular ferrite in the austenite matrix were observed in the as-welded FZ (Figure 1b). Moreover, the cold-rolled FZ showed a lesser degree of hardening (306 HV0.3) than the 304L-BM (340 HV0.3). The cold-rolled FZ had fewer slip bands (Figure 1c) than that of the 304L-CR specimen. Recovery during sensitization treatment led to a decrease in hardness to 261 HV0.3 for the 308L-CRS specimen. Sensitization of cold-rolled 308L deposit also enhanced the Cr-rich carbides to precipitate in the interfaces between the δ-ferrite and transformed austenite matrix, which were confirmed by EDS analysis (Figure 1d) and TEM examinations (Figure 1e). Similar results were observed in the sensitized 304L weld after cold rolling [31].

**Figure 2:**
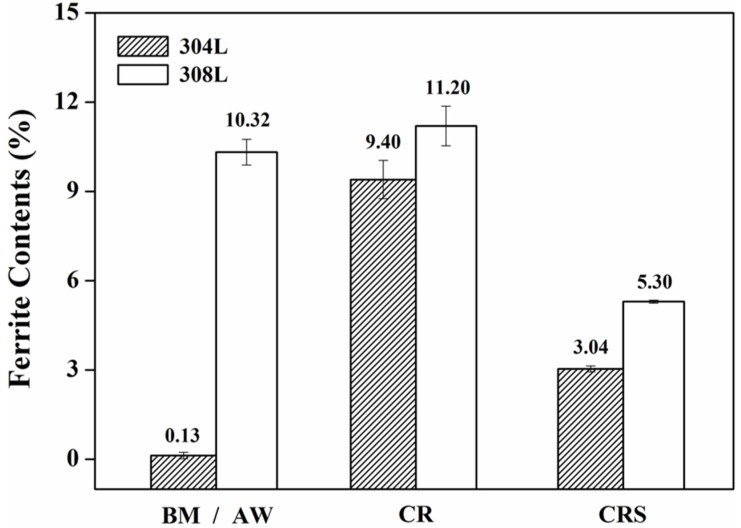
The ferrite contents of the 304L substrates and 308L deposits in distinct conditions.

Figure 2 shows the ferromagnetic phase in the specimens as determined by the ferritescope, which could be either the δ-ferrite formed in the solidified structure in the FZ or the induced α′-martensite in the specimen after cold rolling. The ferromagnetic phase content of the 304L substrate (304L-BM), which had been subjected to solution treatment at 1050 °C/0.5 h, was rather low (0.13%). The as-welded 308L deposits (308L-AW) consisted of 10.32% ferrite, which could effectively prevent hot cracking of the FZ. Cold-rolling the 304L substrate (304L-CR) induced the formation of α′-martensite content of 9.4%; however, a minor increase in ferromagnetic phase content (by less than 1.0%) was observed in the weld deposits after rolling (308L-CR). This implied that the induced α′-martensite was less likely to be formed in the 308L deposit than in the 304L substrate. Sensitization caused about 6.4% of the induced α′-martensite in the cold-rolled 304L to revert to austenite (304L-CRS). It was clear that induced α′-martensite in the 304L was very sensitive to heating at elevated temperatures. Furthermore, the ferromagnetic phase content was reduced to approximately 5.3% in the sensitized 308L deposit (308L-CRS). The result indicated that some of the δ-ferrite was also transformed into austenite during the sensitization treatment. It is pointed out that strain-induced martensite increases with tensile pre-strain of 304 SS, while more carbides precipitate intragranularly in the strained specimens after sensitization at 948 K for five hours [32]. Such a significant change in microstructure was expected to cause a marked decline in corrosion resistance.

### 3.2. Electron Backscatter Diffraction (EBSD) Analysis

**Figure 3:**
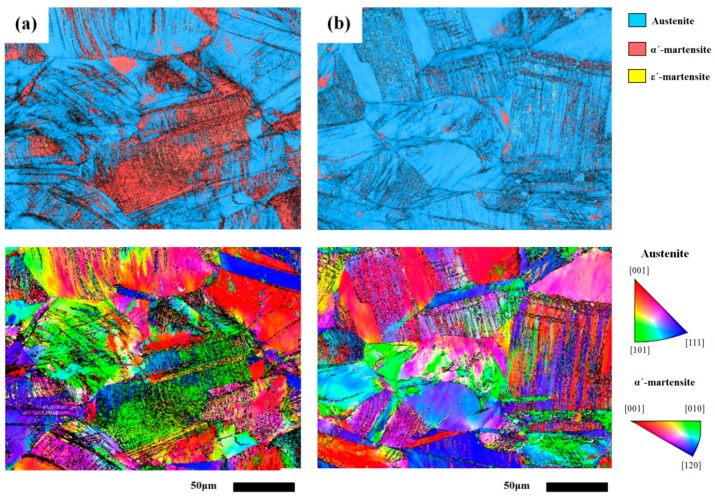
The Electron Backscatter Diffraction (EBSD) image (top) and the corresponding inverse pole figure IPF (Inverse pole figure) (bottom) of (**a**) 304L-CR and (**b**) 304L-CRS specimens.

**Figure 4:**
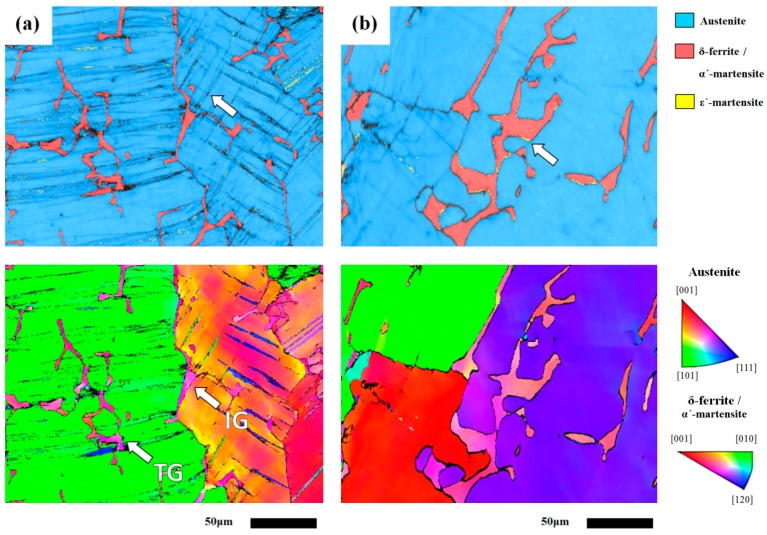
The EBSD image (top) and the corresponding IPF (bottom) of the (**a**) 308L-CR and (**b**) 308L-CRS specimens.

Figure 3 and Figure 4 are the EBSD maps showing different microstructures in the cold-rolled 304L steel plate or 308L weld overlay, with or without sensitization treatment. For the ease of inspecting the induced martensite in the samples after cold rolling, the samples were subjected to cold rolling with a thickness reduction of 30%. The results indicated that cold rolling caused the transformation of austenite to marteniste (Figure 3a-top). The α′-martensite was more likely to be formed in the intense slip regions. With a cold rolling of a 30% reduction in thickness, the as-rolled 304L showed a slight distortion of grain profile and the occurrence of multiple slips in different grains, as shown in Figure 3a-bottom. Sensitization treatment led to a significant decrease in the amount of α′-martensite (Figure 3b-top), which was confirmed by the measurements of ferrite content. Moreover, tortuous slip bands were replaced by straight ones in the cold-rolled 304L after sensitization. In addition, the grain boundaries of the sensitized specimen could be defined more clearly relative to the as-rolled one (Figure 3b-bottom).

All 308L weld-metal specimens were wire-cut from the overlay welds, and subjected to rolling with/without sensitization treatments. The EBSD map is reported to be able to identify the inherent microstructure and orientation in distinct regions of a 304L weld deposited by 308L filler metal [33]. As shown in Figure 4a-top, cold rolling only caused the formation of sparse slip bands in the as-rolled 308L deposit. Additionally, the EBSD map could be used to distinguish the grain orientation and the location of δ-ferrite in the 308L deposit (Figure 4a-bottom). The results revealed that skeletal δ-ferrite could be formed in the deposit intra-(TG) and intergranularly (IG), not all of the δ-ferrite was located at the dendrite boundaries. With the sensitization treatment, the reversion of δ-ferrite and martensite to austenite accounted for the decrease in ferrite contents of the specimen. The great reduction in slip bands together with the ε-martensite located at the interfaces between the δ-ferrite and γ matrix was obtained in the 308L-CRS specimen, as indicated by the arrow in Figure 4b-top.

### 3.3. Weight-Loss Tests in a Salt Spray

**Figure 5:**
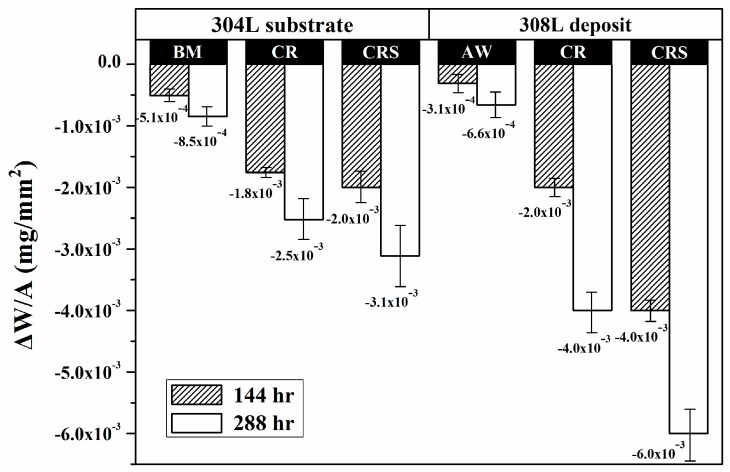
The weight loss of the 304L substrates and 308L deposits in distinct conditions after exposure to a salt spray.

Figure 5 shows the weight loss per unit area of the specimens, which was used to evaluate the corrosion resistance in a salt spray. The results indicated that the sensitized specimens had the highest weight loss of all the specimens of each group. The 304L-BM and the 308L-AW specimens were more resistant to saline corrosion in each group. The 308L-AW specimen had slightly less weight loss than the 304L-BM, possibly due in part to the higher Cr and Ni contents of the former. The results also indicated that rolling caused an obvious increase in weight loss and sensitization made it worse, especially for the 308L deposits. The 308L-CRS specimen had the greatest weight loss of the tested specimens. Cold work was harmful, and sensitization treatment after cold rolling was quite detrimental to the corrosion resistance of the specimens in a salt spray, regardless of the specimen.

### 3.4. U-Bend Tests in a Salt-Spray Environment

**Figure 6:**
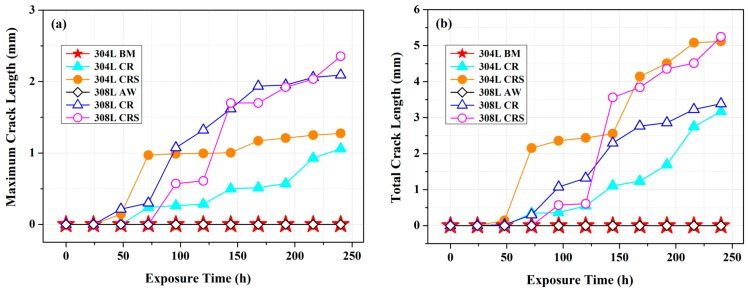
The variation in crack length of the U-bend specimens vs. the testing period in the salt-spray environment: (**a**) maximum crack length; and (**b**) total crack length.

Figure 6 shows the variations in the maximum crack length (MCL) and total crack length (TCL) of the U-bend specimens as a function of the exposure time to a salt spray. The 304L-BM and 308L-AW specimens had a lack of visible cracks over the testing period, indicating that they were more resistant to SCC in a salt spray than the other specimens in each group. Regarding the MCL (Figure 6a), the longest crack lengths of the 308L-CR and 308L-CRS specimens were similar at the end of the test. The difference between them was the shorter incubation time and initial faster crack growth rate of the former. Overall, the cracking susceptibility of the 308L deposit was higher than that of the 304L substrate under the same specimen-preparation conditions. The 304L-CR specimen was expected to have higher residual tensile stress than the 304L-CRS specimen, that higher stress levels were expected to increase the SCC susceptibility. However, the MCL of the 304L-CR specimen was obviously shorter than that of the 304L-CRS specimen if the testing period was shorter than 200 h. Inevitably, the sensitization increased SCC susceptibility of the cold-rolled 304L substrate. 

Figure 6b shows the SCC susceptibility of distinct U-bend specimens ranked according to their TCLs. In each group, the CRS specimen was more sensitive to SCC than the un-sensitized specimens. The 304L-CRS and 308L-CRS specimens seemed to have equivalent SCC susceptibility in a salt spray. The difference between them were cracks tending to emerge in the 304L-CRS specimen shortly after the start of testing. Despite of ranking methods, the ease of material dissolution at the slip bands and grain boundaries, which were available crack nucleation sites, accounted for a shorter crack incubation period of the 304L-CRS specimen than the other specimen (discussed later in the text). Furthermore, the TCLs of the 308L-CR and 304L-CR specimens were also similar at the end of the test. Regarding the cracking susceptibility of the 308L-CR and 304L-CR specimens, the difference in character between them was that the longest crack grew much faster in the 308L-CR than that in the 304L-CR specimen, as shown in Figure 6a. Furthermore, the TCLs of the 308L-CR specimen were longer than those of the 304L-CR while testing within 200 h. The presence of greater residual tensile stress in the 308L-CR specimen might play an important role for such differences between them.

### 3.5. Surface Features of Weight Loss and U-Bend Specimens

**Figure 7:**
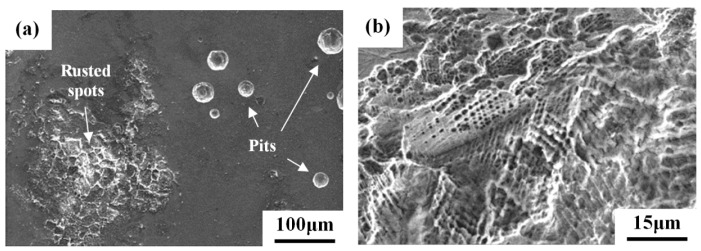

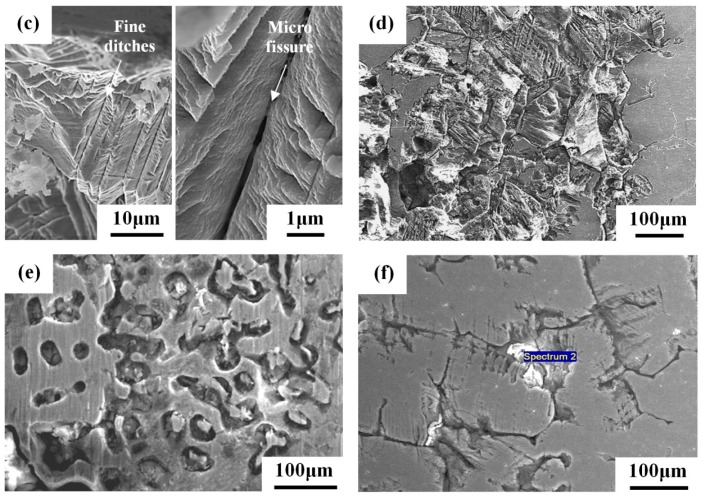
SEM micrographs showing the surface morphologies of weight-loss specimens: (**a**) 304L-BM; (**b**,**c**) 304L-CR; (**d**) 304L-CRS; (**e**) 308L-AW; and (**f**) 308L-CR specimens.

The damaged surface morphologies of weight-loss specimens examined by SEM are shown in Figure 7. The results indicated that round surface pits were more likely to occur in the 304L-BM specimen (Figure 7a). Agglomeration of individual pits formed rusted spots on the surface of the 304L-BM specimen. In the 304L-CR specimen, surface damage preferentially selected the area with intense slip bands (Figure 7b). The intersections of slip bands corroded more severely and formed micro-pores therein. In addition to the surface corrosion, severe corrosion at the slip bands formed fine ditches, which were likely to turn into micro-fissures cutting into the specimens (Figure 7c). The surface features of the 304L-CRS specimen exhibited severe corrosion at the slip band, as with the 304L-CR specimen, and induced the growth of fine cracks along austenite grain boundaries (Figure 7d). In contrast, the metal dissolved preferentially at the interfaces between the skeletal structures and the austenite matrix, and some residual debris remained in the solidified structures in all of the 308L deposits (Figure 7e). Thus, metal dissolution at the interfaces of the skeletal structures of all the 308L deposits at different levels was responsible for the weight loss of the specimens in a salt spray. In addition to corrosion along the solidified boundaries, the slip bands in the 308L-CR specimen also corroded preferentially, just as what occurred in the 304L-CR specimen, as shown in Figure 7f. Furthermore, the large spall area in the solidified boundaries of the 308L-CRS specimen led to it having the highest weight loss of the specimens being tested.

**Figure 8:**
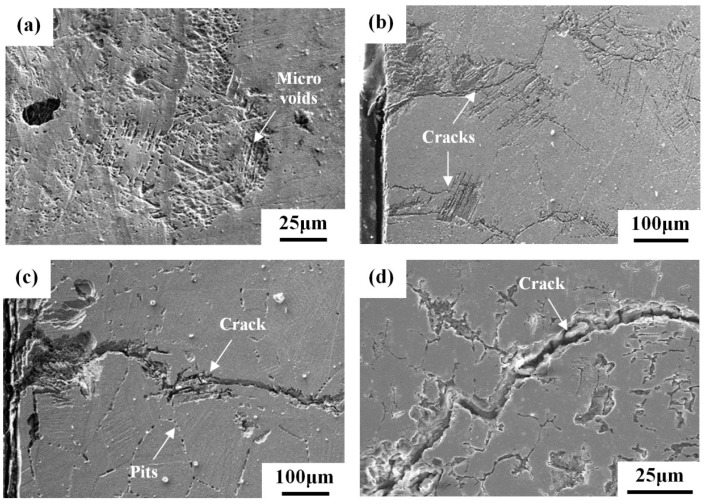
SEM micrographs showing the surface morphologies of U-bend specimens: (**a**) 304L-BM; (**b**) 304L-CR; (**c**) 304L-CRS; and (**d**) 308L-CRS specimens.

With the applied five percent strain in the U-bend specimens, the occurrence of deformation would introduce slip bands into the 304L-BM and 308L-AW specimens. A small sample was cut from the U-bend specimen and subjected to metallographic preparation for further inspection of the SCC cracks. Figure 8 shows the superficial crack path of the U-bend specimens after salt spray. The slip bands of the 304L-BM specimen assisted the formation of oriented microvoids (Figure 8a). The linking of microvoids in the 304L-BM specimen was expected to turn into microcracks if the testing interval had been prolonged. For the 304L-CR specimen, cracks initiated at the slip bands and might propagate parallel or transverse to the slip bands (Figure 8b). Stepwise crack growth was seen in the prior work studying the SCC of 304L in the salt-spray environment [6]. In the case of the 304L-CRS specimen, the cracks tended to initiate at the slip band and then might propagate along the prior austenite grain boundaries (Figure 8c). Furthermore, corrosion pits at the grain boundaries were more likely to be seen in the 304L-CRS specimen. Intergranular corrosion seemed to promote intergranular SCC of the 304L-CRS specimen. The surface morphology of the 308L deposit in the U-bend test was similar to that of its counterpart specimen in the weight-loss test. All 308L deposits exhibited crack growth mainly along the skeletal structure, irrespective of the specimen preparation (Figure 8d). Therefore, the dissolution of the skeletal structure to different degrees was the main cause of SCC of the 308L deposits. The large weight loss of the 308L-CRS specimen was confirmed by severe spalling of the skeletal structure in Figure 8d and was related to its high SCC susceptibility.

**Figure 9:**
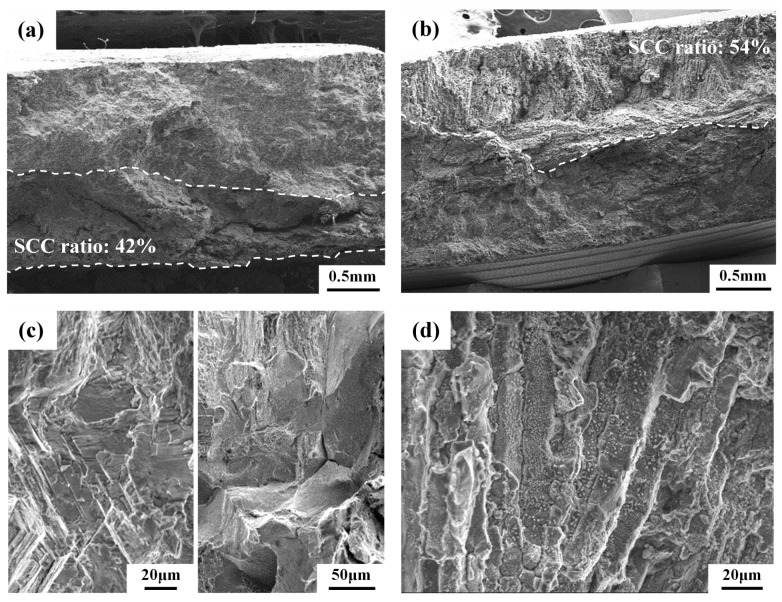
The SEM fractographs of the U-bend specimens after exposure to salt spray: macroscopic features of the (**a**) 304L-CRS and (**b**) 308L-CR specimens; microscopic features of the (**c**) 304L-CR (left) and 304L-CRS (right); and (**d**) 308L-CR specimens.

Figure 9 presents SEM micrographs showing the typical fracture morphology and the crack path of the U-bend specimens. After salt-spray tests, assorted specimen sizes were sliced from the tested specimen and then bent into two pieces after immersion in liquid nitrogen for a few seconds. Figure 9a,b reveal the halves of the broken specimens symmetrical to the centerline of the drilled hole. The SCC ratio is defined as the SCC region over the fracture surface inspected by SEM. The macroscopic fracture appearances of the 304L-CR and 304L-CRS specimens were similar (Figure 9a), showing mainly transgranular fracture at the crack initiation stage. Moreover, the fracture appearances of the 308L-CR and 308L-CRS specimens were also alike, showing the features of an elongated solidified structure (Figure 9b). Inspection of the fracture surfaces of the U-bend specimens at higher magnifications by SEM, transgranular cracks initiated and propagated along the slip bands in the 304L-CR specimen (Figure 9c left), and mainly transgranular fractures mixed with fewer intergranular fractures were observed in the 304L-CRS specimen (Figure 9c right). In addition, the stress corrosion cracks of the 308L-CR and 308L-CRS specimens propagated along the solidified boundaries (Figure 9d). It was obvious that the dissolution of the skeletal structure was the cause of the SCC of 308L deposits in a salt spray.

## 4. Discussion

In comparison with the granular structure of the 304L substrate, the dendritic structure consisted of a certain amount of δ-ferrite in the 308L deposit. The EBSD map showed that only a few α′-martensite particles were induced in the intersection zones of slip bands in the 308L deposit, as indicated by the arrow in the Figure 4a-top. Such characters were responsible for the minor increase in ferrite contents of the 308L-CR specimen, in contrast to an obvious rise in the ferrite contents of the 304L-CR one. As shown in Figure 4a-bottom, skeletal δ-ferrite in the 308L deposit could be located intra- and intergranularly. Within one grain, the slip bands were found to cross over the δ-ferrite. Furthermore, multiple slips were less likely to be seen in the deposit. It was deduced that those intragranular δ-ferrites might act as barriers to dislocation slips, resulting in less strain-hardening and martensite formation relative to the 304L substrate.

The deposition of synthetic sea water to simulate the effect of sea salt particles on the SCC of 304 and 304L was investigated by constant load method in the temperature range of 60–80 °C at relative humidity (*RH*) = 35% [3]. The 304 and 304L SSs are very sensitive to chloride-induced SCC [3]. Moreover, in the as-received and sensitized conditions, the 304 and 304L SSs only show corrosion pits if the stress is not applied [3]. In this study, the surface morphology of the weight-loss specimens of the 304L-BM specimen revealed that round surface pits were likely to be found (Figure 8a), and agglomeration of individual pits produced rusted spots. Thus, the present results were consistent with those reported by Mayuzumi et al. [3].

It has been pointed out that preferential dissolution of the slip bands results in the increased SCC susceptibility of cold-worked 304L SS at room temperature in 1 M HCl solution [12]. Moreover, the intersections of slip bands would assist the formation of α′-martensite therein [22,34,35]. As shown in Figure 7b,c, the surface morphology of the weight-loss specimens in a salt spray for the 304L-CR specimen showed preferential dissolution in the area with intense slip bands. The results clearly indicated those intersecting sites tended to dissolve more easily in a salt spray. The weight-loss and U-bend tests proved that the dissolution of slip bands in the cold-worked or strained 304/304L SSs played an important role in crack initiation and propagation in a salt spray. Therefore, the presence of α′-martensite, which was responsible for the formation-oriented micro-pores in the slip bands after salt spray, was detrimental to the corrosion and stress corrosion resistance of 304/304L SSs. It was deduced that any permanent deformation of the 304/304L will inevitably enhance SCC susceptibility in a salt spray.

With salt deposited on the surface, the sensitized 304/304L (650 °C/1 h) has a shorter rupture time than the unsensitized one at 80 °C at *RH* = 35% in the low applied stress region [3]. SEM morphology of the 304L-CRS specimens showed that severe corrosion occurred at the slip band and induced the growth of fine cracks along the austenite grain boundaries (Figure 7d) in the weight-loss tests. Furthermore, mainly transgranular cracks initiated at the slip band might then propagate in an intergranular manner in the 304L-CRS specimen in U-bend tests (Figure 9c). In prior studies, the cracks propagating along the austenite grain boundaries of sensitized 304L SS are associated with the formation of α′-martensite therein as the cracks grow [6,36]. As shown in Figure 6, the CRS specimen was more sensitive to SCC than the other specimens in each group. The CRS specimens were also known to have the highest weight loss of the specimens in each group (Figure 5). Therefore, the metal dissolution at the slip bands, austenite grain boundaries, or δ-ferrite/austenite interfaces in the CRS specimens were responsible for the crack initiation of SCC in a salt spray. Thus, the high SCC susceptibility of the 304L-CRS SS and 308L-CRS deposits are associated with the high weight loss of the alloy in a salt spray.

Droplets of saturated chloride salt solution are deposited on the U-bend specimens to investigate the chloride-induced SCC of austenitic and duplex SSs in an environmental chamber (20–50 °C, 30%–70% *RH*) for 10 weeks [5]. The degradation of duplex SSs by CaCl_2_ or MgCl_2_ salt deposits at low *RH* is due to the selective corrosion of ferrite phase [5]. The interdendritic corrosion of the fusion zone of a 304 spot weld is associated with the corrosion attack of δ-ferrite [37]. In a prior study [29], the dissolution of the skeletal structures in all 308L and 309LMo deposits were responsible for the crack initiation and growth of the U-bend specimens in a salt spray containing 10 wt % NaCl at 120 °C [29]. In this work, the high cracking sensitivity to SCC of the 308L-CRS specimens was the result of the corrosion-induced crack initiation and growth along the skeletal structure. Therefore, the SCC susceptibility of the 308L deposits could be associated with the weight-loss results of the deposit. As a whole, the SCC susceptibility of the investigated specimens in a salt spray was related to their weight-loss characteristics.

## 5. Conclusions

1. The 308L deposit consisted of a certain amount of δ-ferrite distributed intra- and intergranularly in the solidified structure, as compared with the granular structure of the 304L substrate. With the same thickness reduction after cold rolling, the 308L deposit showed a lesser tendency of transformation of austenite to martensite. Those intragranular δ-ferrites in the 308L deposit might act as barriers to dislocation slips, resulting in less strain-hardening and martensite formation relative to the 304L substrate.
2. The results of the weight-loss tests revealed that the sensitization treatment was very harmful to the corrosion resistance of the specimens in salt spray, irrespective of the groups of the 304L substrate and 308L deposit. The dissolution of the skeletal structures in all 308L deposits at various levels was responsible for the high weight loss of the specimens.
3. None of the specimens of the 304L base metal or the as-welded 308L deposit of the U-bend exhibited visible cracks during the testing interval, indicating that these samples were resistant to SCC in a salt spray. Crack initiation within a short time, and rapid crack growth in the 308L-CR specimens after testing, could be attributed to the presence of high residual tensile stress. The ease of dissolution along the skeletal structure of the 308L-CRS specimens, which was confirmed by their high weight loss, accounted for the high SCC susceptibility. Moreover, assisted dissolution at the slip bands enhanced the SCC susceptibility for the specimens subjected to cold rolling. The increased trend of grain boundary corrosion in cold-rolled and sensitized specimens (304L-CRS) resulted in increasing SCC susceptibility. Therefore, the SCC susceptibility of the investigated specimens in a salt spray was associated with their weight-loss characteristics.

## Figures and Tables

**Table 1:** Chemical compositions of the 304L steel plate and 308L weld metals used in this study.

Material	Chemical Composition (wt %)							
	C	Mn	Si	S	P	Cr	Ni	Fe
AISI 304L	0.019	1.53	0.50	0.030	0.030	18.20	8.04	bal.
ER 308L	0.018	1.90	0.32	0.010	0.017	19.70	10.10	bal.

**Table 2:** Test matrix of the current study.

Material	Testing Condition		
	Base Metal (BM)/As-Welded (AM)	Cold-Rolled (CR)	Sensitization after Cold Rolling (CRS)
AISI 304L	304L-BM	304L-CR	304L-CRS
ER 308L	308L-AW	308L-CR	308L-CRS

**Table 3:** Average Vickers-Hardness of the various specimens.

Specimen	Vickers-Hardness (HV0.3)		
	BM/AW	CR	CRS
304L	165	340	278
308L	164	306	261

## List of Abbreviations

SS	Stainless steel
SCC	Stress corrosion cracking
BWR	Boiling water reactor
FZ	Fusion zone
ER	Electrode rod
BM	Base metal
AW	As-welded
CR	Cold-rolled
CRS	Sensitization after cold rolling
MCL	Maximum crack length
TCL	Total crack length
OM	Optical microscope
SEM	Scanning electron microscope
EBSD	Electron backscatter diffraction
TEM	Transmission electron microscope
EDS	Energy dispersive spectroscopy
TG	Intra-granular
IG	Inter-granular
IPF	Inverse pole figure
RH	Relative humidity
